# Is the rate of bile leak higher in clipless laparoscopic cholecystectomy compared to conventional cholecystectomy?

**DOI:** 10.1016/j.amsu.2021.01.038

**Published:** 2021-01-19

**Authors:** Sabry Abounozha, Rashid Ibrahim, Talal Alshahri

**Affiliations:** a Northumbria Healthcare NHS Foundation trust, Northumbria, UK; b University Hospitals Plymouth NHS Trust, Plymouth, UK; c Imam Abdulrahman Alfaisal University Hospital, Riyadh, Saudi Arabia

**Keywords:** Clipless cholecystectomy, Conventional cholecystectomy, Standard cholecystectomy, Vessel sealing devices, Ultrasonic scalpel, Bile leak

## Abstract

A best evidence topic has been constructed using a described protocol. The three-part question addressed was: In patients undergoing cholecystectomy is the clipless laparoscopic cholecystectomy is associated with higher risk of bile leak compared to conventional cholecystectomy?

The search has been devised and 6 studies were deemed to be suitable to answer the question. The outcome assessed was the rate of bile leak in clipless cholecystectomy compared to conventional laparoscopic cholecystectomy. Authors found that the rates of bile leak in clipless laparoscopic cholecystectomy is comparable to conventional technique. Clipless cholecystectomy is feasible and safe.

## 1 Introduction

This BET was constructed using a framework outlined by the International Journal of Surgery [1]. A BET provides evidence-based answers to common clinical questions, using a systematic approach of reviewing the literature.

## 2 Clinical scenario

Laparoscopic cholecystectomy is one of the most common performed procedures worldwide. As a surgical trainee who does different rotations in different hospitals, probably you will operate with different surgeons with different techniques. One of the evolving techniques is clipless laparoscopic cholecystectomy where the division of cystic duct is undertaken using a vessel sealing device (ultrasonic device). Given that one of the concerns adopting this technique is the risk of bile leak, you wonder whether it has a higher risk compared to conventional cholecystectomy. Therefore, you decide to conduct a systematic review to look for a based evidence answer to this question.

## 3 Three-part question

In [patients undergoing cholecystectomy] is [the clipless laparoscopic cholecystectomy] associated with [higher rates of bile leak compared to conventional cholecystectomy]?

## 4 Search strategy

The search was conducted as following:

Embase 1974 to 2020 and MEDLINE® 1946 to November 2020 using the OVID interface.

[clipless cholecystectomy OR ultrasonic cholecystectomy OR vessel sealing device cholecystectomy] AND [conventional cholecystectomy OR standard cholecystectomy] AND [bile leak OR postoperative bile leak]

The search was limited to English language and human studies.

## 5 Search outcome

**Fig. 1:**
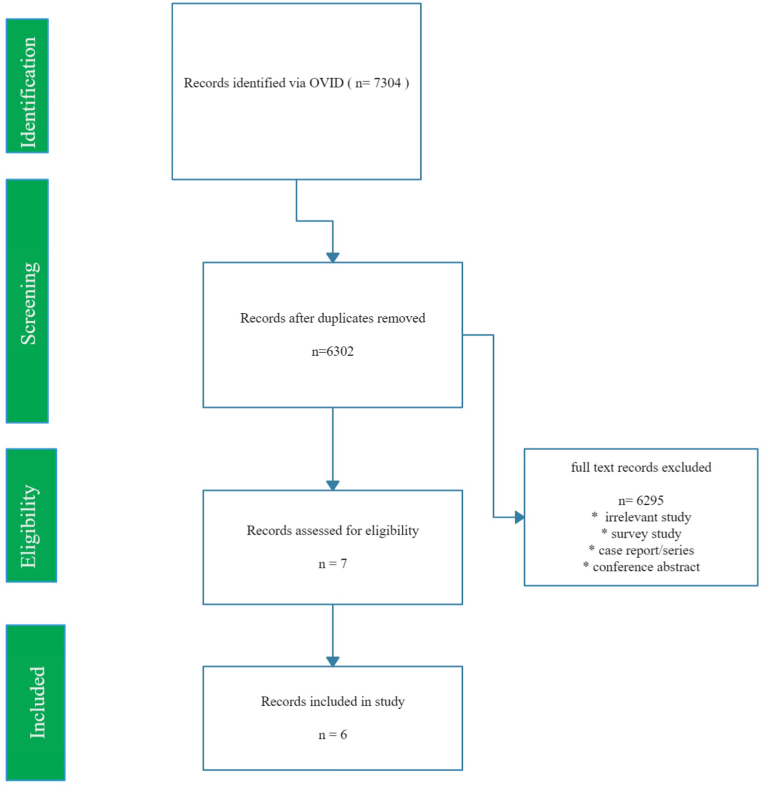
PRISMA Flow chart

7304 articles were found. Out of these 7 deemed to be suitable and met the criteria of our search after removing the duplicate and excluding the irrelevant articles. 6 out of 7 articles were chosen as they compared the bile leak rates between the two techniques (Fig. 1).

### 5.1 Exclusion criteria

1 Studies not comparing both techniques
2 Conference abstracts
3 Low evidence papers
4 Absence of full-text articles

## 6 Result

(please refer to the Table 1)

**Table 1:** Result

Author, date of publication, journal and country	Study type and level of evidence	Patient group	Outcomes	Follow up	Key results	Additional comments
Bessa et al., 2008, Journal of Laparoendoscopic & Advanced Surgical Techniques, Egypt	Randomized Controlled Study, Level II	120 patients with symptomatic gallstone disease were randomly assigned to either the harmonic scalpel laparoscopic cholecystectomy group (HS group = 60 patients) where closure and division of the cystic duct was achieved solely by the harmonic shears or the clip and cautery laparoscopic cholecystectomy group (C&C group = 60 patients)	To compare between the safety and efficacy of the harmonic shears and the commonly used clip and cautery technique in achieving safe closure and division of the cystic duct in the laparoscopic cholecystectomy	Both groups were followed-up for 6 months	Neither minor nor major bile leaks were encountered in either group. The harmonic shears are as safe and effective as the commonly used clip and cautery technique in achieving safe closure and division of the cystic duct in the laparoscopic cholecystectomy	Single centre, no power calculation, no blinding was mentioned, Patients with common bile-duct stones, acute cholecystitis, previous upper abdominal operation, suspicion of gallbladder malignancy and pregnant patients were excluded, risk of bias cannot be excluded
Kandil et al., 2009, J Gastrointest Surg, Egypt	Randomized Controlled Study, Level II	This study included group A (70 patients) in whom LC was conducted using the traditional method (TM) by clipping both cystic duct and artery and dissection of gallbladder from liver bed by diathermy, and group B (70 patients) LC was conducted using harmonic scalpel (HS) closure and division of both cystic duct and artery and dissection of gallbladder from liver bed by HS	to compare the traditional method of laparoscopic cholecystectomy (LC) versus LC using harmonic as regard to bile leak	Both groups were followed-up for 6 months	HS provides a complete biliary stasis and is a safe alternative to stander clip of cystic duct and artery. No statistical difference in intraoperative and postoperative bile leak. Intraoperative bile spillage was 13(18.6%) in group A and 5 (7.1%) in group B (P = 0.04). Postoperative bile leakage was 2 (2.9%) in group A and 0 in group B (P = 0.156)	Single Centre, no power calculation, no blinding was mentioned, patients above 80 years old, patients with history of upper laparotomy, patients with common bile duct stones and pregnant women were excluded, risk of bias cannot be excluded
Nakeeb et al., 2010, Surg. Endoscopy Journal, Egypt	Randomized Controlled Study, Level II	Group A (60 patients) underwent LC by the traditional method (TM) with clipping of both the cystic duct and artery and dissection of the gallbladder by diathermy, and group B (60 patients) had LC performed using Harmonic scalpel (HS) closure and division of both the cystic duct and artery with dissection of the gallbladder by the HS	This study aimed to compare the traditional method for LC with LC using the Harmonic scalpel in terms of safety and bile leak for cirrhotic patients	Both groups were followed-up for 6 months	The Harmonic scalpel provides complete biliary stasis and is a safe alternative to the standard clipping of the cystic duct. Bile leak was encountered in 1.7% with HS and 3.3% with TM (p = 0.45)	Single Centre, no power calculation, no blinding was mentioned, patients older than 80 years, patients with a history of upper laparotomy, patients with common bile duct stones, patients with decompensated liver disease, and pregnant women were excluded, risk of bias cannot be excluded
Jain et al., 2011, Journal of Laparoendoscopic & Advanced Surgical Techniques, India	Randomized Controlled Study, Level II	200 patients with symptomatic gallstone disease, randomly divided into two groups (100 each), one undergoing cholecystectomy using ultrasonically activated shears and the other using conventional clip and electrocautery	to compare the traditional method of laparoscopic cholecystectomy (LC) versus LC using harmonic as regard to bile leak	Both groups were followed-up for 6 months	Ultrasonically activated scalpel can be used safely in laparoscopic cholecystectomy without risk of major injuries or leaks. There was no incidence of bile leak during a 6-month follow-up period in either of the groups	Single Centre, randomization process is not clear, no power calculation, no blinding was mentioned, patients above 70 years old, impaired liver function tests, history of jaundice or pancreatitis, suspicion of gallbladder carcinoma, patients having concomitant common bile duct (CBD) calculi, acute cholecystitis, cholangitis, and empyema of gallbladder, pregnant patient, CBD size more than 5 mm on ultrasonography were excluded, risk of bias cannot be excluded
Wills et al., 2013, Journal of Laparoendoscopic & Advanced Surgical Techniques, USA	Retrospective cohort study, Level III	208 patients received surgical clip placement or the Harmonic scalpel was used for cystic duct occlusion. Surgical clips used in 148 patients and Harmonic scalpel 57 patients.	To compare the bile leakage rates in both groups	Not mentioned	The use of the Harmonic scalpel is deemed safe and comparable to clip placement at the discretion of the surgeon for cystic duct ligation. The use of the Harmonic scalpel versus clip placement had comparable rates of bile leak at 1.75% and 0.66%, respectively	Small sample size, large discrepancy between the number of participants in each group, harmonic scalpel cystic duct closure was used only for cystic ducts less than 5 mm in diameter while stapler used for larger diameters, risk of bias cannot be excluded
Sanawan et al., 2017, Journal of the College of Physicians and Surgeons Pakistan, Pakistan	Randomized controlled study, Level II	150 cases (75 in each group) were randomized into two groups, harmonic scalpel clipless group (HSG) versus conventional laparoscopic cholecystectomy (CLC) with electrocautery group	To determine the efficacy of ultrasound shear in laparoscopic cholecystectomy in terms of postoperative bile leaks	All patients were followed-up for 4 weeks	None of the patients in either group had bile leaks	Single Centre, power calculation undertaken, follow up was for 4 weeks only, common bile duct stones, intrahepatic biliary channel dilatations, raised gamma GT or alkaline phosphatase (evidence of obstructive jaundice), fever with rigors and chills, previous hepatobiliary surgery, and previous midline abdominal surgeries were excluded, patients with cystic duct diameter more than 5 mm were excluded, risk of bias cannot be excluded

## 7 Discussion

In 2008, Bessa et al. [2] conducted a randomized controlled study comparing clipless laparoscopic cholecystectomy versus conventional technique in terms of safety and bile leak. The authors included 120 patients with symptomatic gallstone disease who were randomly assigned to either the harmonic scalpel laparoscopic cholecystectomy group (HS group = 60 patients) where closure and division of the cystic duct was achieved solely by the harmonic shears or the clip and cautery laparoscopic cholecystectomy group (C&C group = 60 patients). They have reported no minor or major bile leaks in either groups and concluded that harmonic shears are as safe and effective as the commonly used clip and cautery technique in achieving safe closure and division of the cystic duct in the laparoscopic cholecystectomy.

In 2009, Kandil et al. [3] devised a randomized controlled trial. The study included 140 patients who were randomized into two groups. Group A included 70 patients in whom laparoscopic cholecystectomy was conducted using the traditional method by clipping both cystic duct and artery and dissection of gallbladder from liver bed by diathermy. Group B included 70 patients where laparoscopic cholecystectomy was conducted using harmonic scalpel. Closure and division of both cystic duct and artery and dissection of gallbladder from liver bed by harmonic scalpel. They have found no statistical difference in intraoperative and postoperative bile leak between the two groups. Intraoperative bile spillage was 13(18.6%) in group A and 5 (7.1%) in group B (P = 0.04). Postoperative bile leakage was 2 (2.9%) in group A and 0 in group B (P = 0.156). The authors concluded that clipless cholecystectomy provides a complete biliary stasis and is a safe alternative to the standard cholecystectomy.

In 2010, Nakeeb et al. [4] conducted a similar study which included 120 patients. They found that bile leak was encountered in 1.7% with clipless cholecystectomy compared to 3.3% with traditional cholecystectomy (p = 0.45). The authors concluded that the Harmonic scalpel provides complete biliary stasis and is a safe alternative to the standard clipping of the cystic duct.

Jain et al. [5] in 2011 conducted another randomized controlled trial which included 200 patients and there was no incidence of bile leak during a 6-month follow-up period in either of the groups.

Wills et al. [6] in 2013 conducted a retrospective cohort study which included 208 patients. Out of these 57 were done using clipless technique. They found that the use of the Harmonic scalpel versus clip placement had comparable rates of bile leak at 1.75% and 0.66%, respectively.

Lastly in 2017, Sanawan et al. [7] conducted a randomized controlled trial which included 150 patients who were randomized into two groups. Half of them underwent clipless cholecystectomy and the other half underwent conventional cholecystectomy. The authors found that none of the patients in either group had bile leaks.

The observed limitation to all of the abovementioned studies is the risk of bias.

## 8 Clinical bottom line

All of the abovementioned studies have found comparable rates of bile leaks between clipless and conventional laparoscopic cholecystectomy. Therefore, it appears that the clipless laparoscopic cholecystectomy is a safe and feasible technique.

## Ethical approval

Not applicable.

## Sources of funding

None.

## Declaration of competing interestCOI

None.
